# The Emerging Tool of the Human Anellovirome

**DOI:** 10.3390/v16060990

**Published:** 2024-06-20

**Authors:** Daniele Focosi, Pietro Giorgio Spezia, Fabrizio Maggi

**Affiliations:** 1 North-Western Tuscany Blood Bank, Pisa University Hospital, 56124 Pisa, Italy; daniele.focosi@gmail.com; 2 Laboratory of Virology, National Institute for Infectious Diseases Lazzaro Spallanzani-IRCCS, 00149 Rome, Italy; fabrizio.maggi@inmi.it

The blood virome is dominated by the Anelloviridae family, which emerges early in life; the anellome, which represents the variety of anelloviruses within an individual, stabilizes by adulthood. Torquetenovirus (TTV) is a paradigmatic example of how an orphan virus can have an impact on modern medicine. While no specific disease has been linked to primary infection, its very high prevalence in the general population has made TTV useful as a biomarker. Since the end of the first decade of the 2000s, a series of studies have identified TTV viremia as a reliable indicator of functional immune competence across various immunocompromised patient populations, especially hematopoietic stem cell and solid organ transplant recipients, where TTV load measurement may help in optimizing immunosuppressive medication dosing.

This Special Issue introduces several innovative applications for TTV. Our group optimized a manual real-time PCR (previously developed in our laboratories) to detect TTV DNA via the Hologic Panther Fusion® System, with this largely improving the turn-around times. The assay, with a detection limit of 1.6 log copies per ml of serum, was validated on clinical samples and showed a 93% concordance rate with a manual PCR assay previously developed in our laboratories [1].

In addition to this, three studies dealt with TTV diversity and next-generation sequencing methods. Researchers from Johns Hopkins and Yale Universities [2] presented a metagenomic sequencing protocol based on rolling circle amplification (RCA) that is tailored for circular single-stranded DNA genomes, using the long-read Oxford Nanopore platform. This is a significant improvement, considering the limitations of current metagenomic methods when dealing with low viral loads of non-linear viruses. Laubscher et al. at the University of Geneva introduced SCANellome, a user-friendly tool to investigate the primate anellome composition at the genus, species, and genotype levels of samples from metagenomics data generated by the Illumina and Nanopore platforms [3]. Finally, in Argentina, Reyes et al. determined TTV species diversity and variability in 27 renal transplant recipients, finding that the median number of TTV species per sample was five, with TTV3 and TTV13 in 75% of cases. No significant correlation between the number of species and viral load was found, but the number and type of TTV species showed complete variability over time [4]. Such heterogeneity may seem detrimental, but so far, universal PCR has shown high correlation rates with clinical correlates. 

Additionally, the University of Groninger provided two studies. Jonker et al. analyzed TTV loads and HDL parameters in serum samples collected at least one-year post-transplantation from 656 stable outpatient kidney transplant recipients, finding a negative association between total HDL particle concentration and TTV load in the non-smoking population and an association between small HDL particle concentration and TTV load [5]. Although causal evidence on the effects of HDL on the immune system remains unknown, these findings open the door for intriguing studies. Doorenbos et al. found that, in addition to cyclosporin and tacrolimus, current smoking and alcohol intake of >20 g/day were positively associated with TTV serum load among 666 stable kidney transplant recipients at ≥1 year after transplantation, suggesting a need to consider smoking status and alcohol intake when applying TTV-guided immunosuppression [6].

Furthermore, two studies investigated TTV kinetics during other acute viral infections. Timmermann et al. at the University of Amsterdam found a significant decrease in anellovirus load in the first weeks after SARS-CoV-2 infection among 20 unvaccinated healthcare workers, whereas anellovirus concentrations remained stable in the uninfected control group. It took about 10 weeks to restore the anellovirus load [7]. In Rome, Abbate et al. with our research group at the National Institute for Infectious Diseases found higher TTV loads in 21 people with acute HIV infection (AHI) than in 13 healthy controls, but this was lower in 28 individuals chronically infected with HIV. During immune reconstitution following antiretroviral therapy, a transient increase in TTV DNA levels was associated with a significant perturbation of activation and senescence markers on CD8 T cells. TTV loads were positively correlated with the expansion of CD8 effector memory and CD57+ cells [8]. These results are consistent with what is understood to be the immune control of TTV; the polyclonal lymphocyte activation and inflammation that accompanies acute infection suppresses TTV replication, which is restored after the immune response subsides.

In conclusion, this Special Issue hosts a review of TTV kinetics in hematological oncology patients—one of the very first cohorts where the role of TTV as a surrogate marker of functional immune competence first emerged [9]. This review highlights its heterogeneity in design, patient characteristics, time points selected for TTV DNA load monitoring, PCR assays used for TTV DNA quantification, and clinical outcomes, which are often poorly defined. Furthermore, the authors recommend prospective randomized controlled trials to elucidate whether TTV DNA load monitoring in the blood may be of any clinical value, with this suggested to be undertaken in a way that is similar to how kidney transplantation is conducted in the framework of the ongoing, Horizon2020-funded TTV-GuideIT trial [10].

## References

[1] Spezia P.G., Carletti F., Novazzi F., Specchiarello E., Genoni A., Drago Ferrante F., Minosse C., Matusali G., Mancini N., Focosi D. (2024). Torquetenovirus Viremia Quantification Using Real-Time PCR Developed on a Fully Automated, Random-Access Platform. Viruses.

[2] Anantharam R., Duchen D., Cox A.L., Timp W., Thomas D.L., Clipman S.J., Kandathil A.J. (2024). Long-Read Nanopore-Based Sequencing of Anelloviruses. Viruses.

[3] Laubscher F., Kaiser L., Cordey S. (2023). SCANellome: Analysis of the Genomic Diversity of Human and Non-Human Primate Anelloviruses from Metagenomics Data. Viruses.

[4] Reyes N.S., Spezia P.G., Jara R., Filippini F., Boccia N., García G., Hermida E., Poletta F.A., Pistello M., Laham G. (2024). Torque Teno Virus (TTV) in Renal Transplant Recipients: Species Diversity and Variability. Viruses.

[5] Jonker J., Doorenbos C.S.E., Kremer D., Gore E.J., Niesters H.G.M., van Leer-Buter C., Bourgeois P., Connelly M.A., Dullaart R.P.F., Berger S.P. (2024). High-Density Lipoprotein Particles and Torque Teno Virus in Stable Outpatient Kidney Transplant Recipients. Viruses.

[6] Doorenbos C.S.E., Jonker J., Hao J., Gore E.J., Kremer D., Knobbe T.J., de Joode A.A.E., Sanders J.S.F., Thaunat O., Niesters H.G.M. (2023). Smoking, Alcohol Intake and Torque Teno Virus in Stable Kidney Transplant Recipients. Viruses.

[7] Timmerman A.L., Commandeur L., Deijs M., Burggraaff M.G.J.M., Lavell A.H.A., van der Straten K., Tejjani K., van Rijswijk J., van Gils M.J., Sikkens J.J. (2024). The Impact of First-Time SARS-CoV-2 Infection on Human Anelloviruses. Viruses.

[8] Abbate I., Rozera G., Cimini E., Carletti F., Tartaglia E., Rubino M., Pittalis S., Esvan R., Gagliardini R., Mondi A. (2023). Kinetics of TTV Loads in Peripheral Blood Mononuclear Cells of Early Treated Acute HIV Infections. Viruses.

[9] Albert E., Giménez E., Hernani R., Piñana J.L., Solano C., Navarro D. (2024). Torque Teno Virus DNA Load in Blood as an Immune Status Biomarker in Adult Hematological Patients: The State of the Art and Future Prospects. Viruses.

[10] Haupenthal F., Rahn J., Maggi F., Gelas F., Bourgeois P., Hugo C., Jilma B., Böhmig G.A., Herkner H., Wolzt M. (2023). A multicentre, patient- and assessor-blinded, non-inferiority, randomised and controlled phase II trial to compare standard and torque teno virus-guided immunosuppression in kidney transplant recipients in the first year after transplantation: TTVguideIT. Trials.

